# Scalable Fabrication and Actuation of a Human Inspired Hand Through 3D Printed Flexures and Combinatorial Actuation

**DOI:** 10.3389/frobt.2022.878111

**Published:** 2022-06-23

**Authors:** Carlo Bosio, Kai Junge, Josie Hughes

**Affiliations:** ^1^ CREATE Lab, Institute of Mechanical Engineering, EPFL, Lausanne, Switzerland; ^2^ Scuola Superiore Sant’Anna, Pisa, Italy

**Keywords:** manipulation, flexures, soft robot control, tactile sensing, rapid fabrication

## Abstract

The fabrication and control of robot hands with biologically inspired structure remains challenging due to its cost and complexity. In this paper we explore how widely available FDM printers can be used to fabricate complex hand structures by leveraging compliant PLA flexures. In particular, we focus on the fabrication of fingers printed as a single piece with tunable compliance, a multi degree of freedom thumb joint, and sensorized compliant fingertips. To address the challenge of control and actuation, we model the behavior of the flexure joints and propose a new method for control: combinatorial actuation. This control method combines the use of a single continuous actuated tendon per finger with two shared “combinatorial” actuators which act across all fingers. We demonstrate that the fingertip workspace using this method is comparable to fully actuated fingers while using significantly less independent actuators. The proposed approach of fabrication and combinatorial actuation provides a rapid and scalable method of designing and controlling complex manipulators.

## 1 Introduction

The development of complex manipulators to perform tasks requiring human level dexterity remains an open challenge (Kemp et al., 2007). One approach is to mimic the design of a human hand including the structure, material properties and tendon routing (Mattar, 2013; Hughes et al., 2016). However, human hands are mechanically and structurally complex structures utilizing 38 actuators (Kontoudis et al., 2019). This makes the fabrication of bio-inspired hands and the accompanying actuation challenging (Pfeifer et al., 2012). Fabrication can be time consuming and manual resulting in limited repeatability. Likewise, the use of many actuators is not feasible and lacks scaleability. New approaches are needed to simplify the mechanical and actuation complexity while exploiting the demonstrated advantages of bio-inspired hands (Hughes et al., 2018; Gilday et al., 2021).

The goal of this paper is to demonstrate low cost methods to both fabricate and control a bio-inspired hand while achieving a high level of dexterity. Here, low cost refers to costs, time, and complexity associated with the material, fabrication process, and control. For the mechanical structure and fabrication, we will utilise Fused Deposition modelling (FDM) 3D printing and limit the number of components to minimize the cost and complexity of fabrication and assembly. For the controller, we aim to minimize the number of actuators while maximizing the overall fingertip workspace, which corresponds to the degree of dexterity.

Existing approaches for 3D printing hands include multi-material hands with complex joint structures (Yang et al., 2017; Hughes et al., 2018), or printing individual bones or elements which are then assembled manually (Gilday et al., 2021; Min and Yi, 2021). These approaches still have significant complexity in fabrication and assembly, and there are limited methods for integrating sensing. An alternative approach is to print flexures, joints that can flex due to their geometry, despite using stiff material. The printing of flexures has been shown to enable a wide range of motion patterns and capabilities (Lotti and Vassura, 2002), enabled by computational design tools (Megaro et al., 2017). Flexures have been used to develop compliant finger joints by using monolithic designs (Mutlu et al., 2016), waved structures (Hughes and Iida, 2017), and hinge structures (Lotti and Vassura, 2002). These structures provide an elegant method for joint design and fabrication, with large deformations possible and the compliance tunable through geometry. However, despite the apparent simplicity of these joints, they are challenging to model and control. Typically bio-inspired hands (and a number of flexure based compliant hands) utilize tendons actuated by motors/servos which imitates human tendons and muscle structures (Palli et al., 2011). Tendons can be controlled antagonistically and typically a one to one mapping of tendon and actuator is used to achieve position and force control of compliant finger structures (Jeong et al., 2018; Takata et al. 2021). However, this method results in a high number of actuators being required as is not scaleable in terms of actuation complexity. A number of approaches have considered how tendon control can be made more scaleable. This includes exploring the use of muscle synergies (Alessandro et al., 2013) or other adaptive synergies (Grioli et al., 2012). This approaches are highly promising, yet must be tailored to specifically work with or exploit flexure based hands.

**FIGURE 1:**
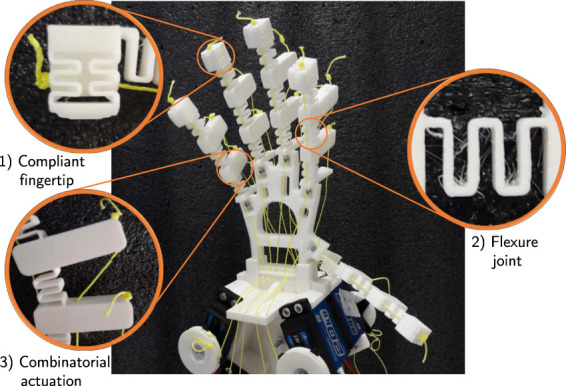
3D printed flexure hand. (1) Compliant fingertip for sensing purposes, (2) geometric shape of the flexure joints, and (3) levers on the back of the finger for combinatorial actuation.

To fully utilise FDM 3D printing to quickly fabricate and assemble a full hand, we propose using flexures to form the finger, thumb, and sensorized fingertip structures (Figure 1). The ability to print a full finger with multiple flexures as a single piece accelerates the fabrication process. The flexures themselves can be modelled and parameterized at the joint level, and their properties can be tuned to maximize the overall workspace of the fingertip. Each finger is actuated by three “tendons”. To further reduce the actuation complexity, propose a new control approach: combinatorial actuation. In this approach, each finger has one independently actuated tendon and two combinatorial “digital” actuators shared across all fingers. The two digital actuators can be set to be either “on” or “off” and can be used combinatorially to vary the workspace of the fingers. By doing so, we are able to reduce the number of actuators required by a hand with n fingers from 3n (since a human finger has 3 joints) to n+2 whilst maintaining the range of possible finger motion (the finger workspace area). As a consequence, we achieve a reduction in actuation complexity. Alternative approaches where each fingers’ moment is decoupled show that a single tendon and actuator can be used per finger (i.e., *n* actuators), however this results in a significant loss of accessible workspace, with only a single trajectory possible. This approach is adopted in a number of works, such as Hussain et al. (2021). A trajectory planner can then be used to control the single independent actuators while switching between different digital states to move around the workspace of each finger.

In this paper we show the capabilities of the flexure based hand for grasping and simple manipulation tasks, and demonstrate combinatorial actuation at both the single finger and whole hand level. We also demonstrate the ability to include fingertip sensing to increase the stability of the grasp. Whilst combinatorial actuation does limit the workspace that can be accessed in static conditions (compared to fully actuated hands), we argue the loss in precision is outweighed by the gains in scalability (in both fabrication and actuation) and reduction in complexity.

In the remainder of this paper we first introduce the methods for the design, modelling, and control of the flexure fingers in Section 2. Following on, the experimental and fabrication procedures are detailed in Section 3. In Section 4 we present the experimental results, and conclude with a discussion in Section 5.

## 2 Materials and Methods

In this section we first present the design and modelling of the tunable flexure based joints and then their integration into full fingers. Using these models we then introduce the combinatorial actuation approach and how trajectories can be planned using it. We finally introduce sensing capabilities that can be integrated into the fingertip.

### 2.1 Flexure Joint

**FIGURE 2:**
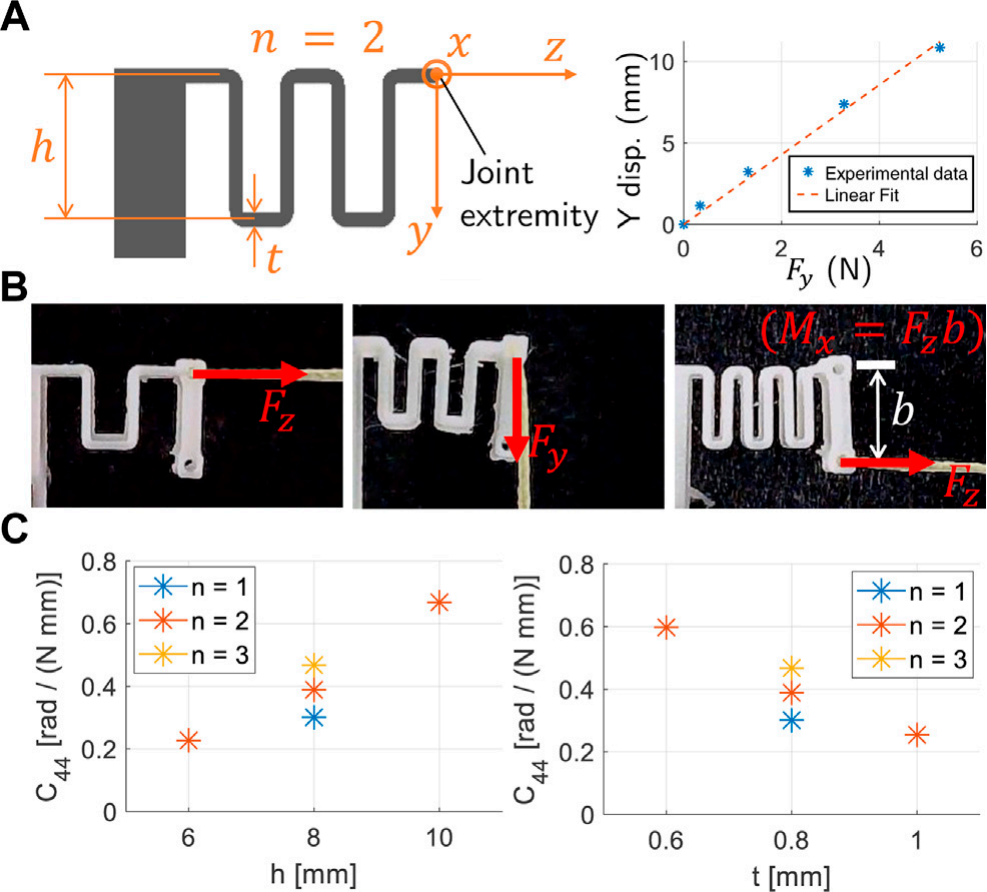
Characteristics of a single flexure joint. **(A)** Diagram of the geometry and the main parameters (*t*: thickness, *h*: height, *n*: number of wave like shapes). Width and length are kept constant (respectively 8 and 15 mm) across all prototypes. Example of linear fitting for the joint deformation, **(B)** Load tests pictures, **(C)** Dependencies of the compliance *C*_44_ (rotation about the *x* axis due to the *M*_*x*_ moment) from the main parameters *t*, *h*, and *n*. In the left plot *h* = 8 mm, in the right plot *t* = 0.8 mm.

To form monolithic 3D printed fingers we require compliant flexure joints to be combined with rigid “bone” sections. Tendons can be routed within the print and actuated to create finger-like motions. The geometry and design of the joints must enable a flexibility and range of motion similar to humans, tunability of the compliance, and ease of printing. The joint design chosen is that of a thin wave like structures, as represented in Figure 2A. This design enables large deformations, has easily tunable compliance, is easy to print, and has a symmetry plane, i.e., the deformation components due to in-plane loads are in-plane as well. The compliance can be controlled by varying a number of geometric properties (Figure 2A): thickness (*t*), height (*h*) and number of waves (*n*).

In order to understand the motion this joint, the load-displacement relationship must be evaluated. Relative to a reference frame on the extremity of the joint (Figure 2A) there are six possible types of loads that can be applied to the structure (forces and moments along the three axes) and six types of deformations (displacements and rotations about the three axes). The joints undergo large displacement, meaning beam theory cannot accurately represent their behavior. We thus adopted an experimental approach to joint characterization. Specifically, we consider applying a force or moment on the joint extremity (see Figure 2A) and describe the resulting displacement. The experimental results demonstrated that, with good approximation, the load-deformation relations are linear (see Figure 2A). This justifies representing the elastic behavior of a single joint between the load and deformation by a linear transformation given by ***δ*** = **C** **F**. Where $F={\left[F_{x},F_{y},F_{z},M_{x},M_{y},M_{z}\right]}^{T}$ (*F*_*i*_ are the forces and *M*_*i*_ are the moments applied to the joint extremity) and $\delta ={\left[\delta_{x},\delta_{y},\delta_{z},\theta_{x},\theta_{y},\theta_{z}\right]}^{T}$ (where *δ*_*i*_ are the displacements and *θ*_*i*_ are the rotations of the extremity), and $C\in R^{6\times 6}$ is the compliance matrix. Since we are only interested in the in-plane motion of the flexure joint, only the highlighted parameters by the red box shown in Eq. 1 relating the in-plane forces and in-plane deformations will be subsequently used.

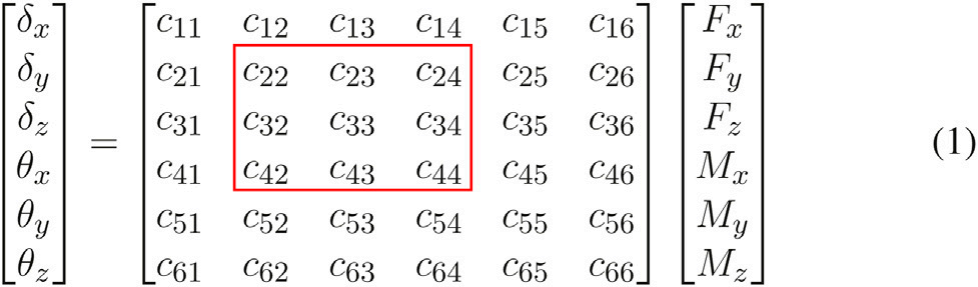

(1)



To analyze the dependency between compliance and joint geometry seven specimens were printed, where we varied each of the three main parameters keeping the other two constant. For these joints we conducted load tests as shown in Figure 2B, measuring the deformation of the joint extremity. From the experimental load-displacement curves we extracted all the relevant compliance values using linear fitting. An exemplar load-displacement plot is shown in Figure 2A. It is important to note that, given the nonlinear nature of the structure (large displacement) and the linear approximation, the compliance matrix defined in Eq. 1 is non-symmetric. An example of the compliances obtained is represented in Figure 2C, with the dependence of the *c*_44_ compliance shown with respect to the thickness *t*, the height *h*, and the number of waves *n*. This shows that with a small change in geometry, the compliance can be varied by over a factor of three, enabling wide customization of the joint properties.

### 2.2 Flexure Finger

**FIGURE 3:**
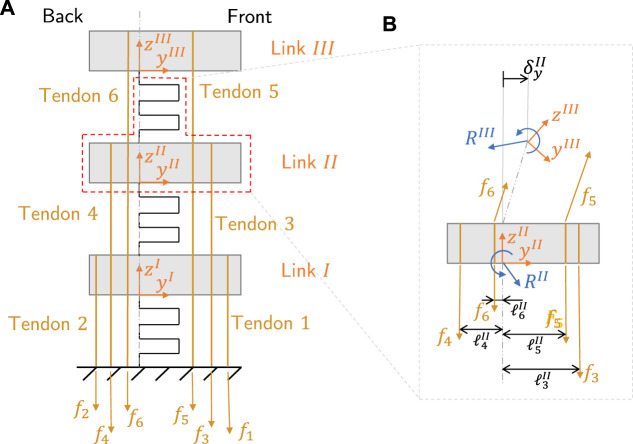
Example of a flexure finger. **(A)** General six tendon diagram with naming conventions for the tendons and links. **(B)** Equilibrium diagram of Link 2. The expression *R* ^*i*^ represents the force and moment constraint reactions on Link *i*, while $\ell_{j}^{i}$ stands for the lever of Tendon *j* when routed through Link *i*. For each link, the deformations of the joint above are relevant for the equilibrium.

A flexure finger is composed by three joints, separated by rigid parts referred to as links. The links are fundamental for tendons routing and for contact interactions with external objects. In the most general case (Figure 3A), a finger can be actuated by six tendons (i.e., two antagonistic tendons per link). Each tendon is attached to a link, and is routed through all links below. For example, Tendon 3 is attached to Link II and goes through Link I). The same tendon can be routed with different moment arms through the links, referred to as levers (*ℓ*). The tendon forces are denoted by *f*_*i*_.

**FIGURE 4:**
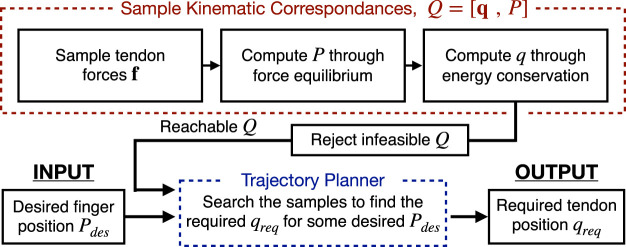
Flowchart of the pipeline to obtain the kinematic correspondences Q = [q P] (red) and then to use the obtained correspondences for trajectory planning (blue).

To be able to control the fingers, we would like to know the kinematic mapping between the fingertip position *P* (in cartesian coordinates) and the corresponding tendon displacement **q** (displacement vector of each attached tendon), which can be actuated by a position controlled servo motor. We denote the pair of *Q* = [**q** *P*] as the kinematic correspondence. The pipeline required to obtain this kinematic correspondence and to use it for trajectory planning (illustrated visually in Figure 4) will be explained in the remainder of this section.

Obtaining the kinematic correspondence is not trivial. As the finger is overconstrained, so an analytical solution is complex to determine. In addition, even if the behavior of the single joints can be linearly approximated, the whole finger structure has a nonlinear behavior where the direction of the load applied to a joint is affected by the displacements of the joints above it.

To begin the process of obtaining *Q*, we sample the tendon forces $f={\left[f_{1},\ldots ,f_{6}\right]}^{T}$ , and $f_{i}∼U\left(0,f_{\max}\right)$ where U is the uniform distribution.

Given some set of tendon force **f**, we can now compute the position of the finger *P*. To do so we must isolate each link from the rest of the system (as exemplified in Figure 3B) such that the reaction forces and moments *R* ^*i*^ (i.e., the internal forces between the link and the joint) can be obtained through force equilibrium. From the reaction force, the joint displacements can be calculated by using the joint model discussed in Section 2.1. The force equilibrium is solved sequentially from link III at the tip to link I. This is because the force equilibrium on link *i* depends on **R** ^*i*+1^. When all three links have been sequentially solved, the relationship between the tendon forces and the fingertip displacements is established.

Finally, we can compute the tendon displacements by using the energy conservation principle (neglecting the contribution of friction) given by:

$$\frac{1}{2}\overset{6}{\underset{j=1}{\sum }}f_{j}q_{j}=\frac{1}{2}\overset{\mathrm{III}}{\underset{i=I}{\sum }}R^{i}\cdot \delta^{i}$$ (2)

where *f*_*j*_ and *q*_*j*_ are the tendons force and displacement, and $R^{i}={\left[F_{y}^{i},F_{z}^{i},M_{x}^{i}\right]}^{T}$ and $\delta^{i}={\left[\delta_{y}^{i},\delta_{z}^{i},\theta_{x}^{i}\right]}^{T}$ are the joints in-plane reaction and deformation vectors.

Now, as illustrated in Figure 4, the kinematic correspondence can be computed. This means for a particular sampled tendon force **f**_**samp**_, we obtain a pair of tendon displacement and fingertip position vector, [**q**_**samp**_ *P*_*samp*_]. From a large number of samples, by rejecting any samples outside the physical limits of the motors used, we obtain a set of samples spanning the configuration space–which can be used to approximate the inverse and forward kinematics of the finger.

**FIGURE 5:**
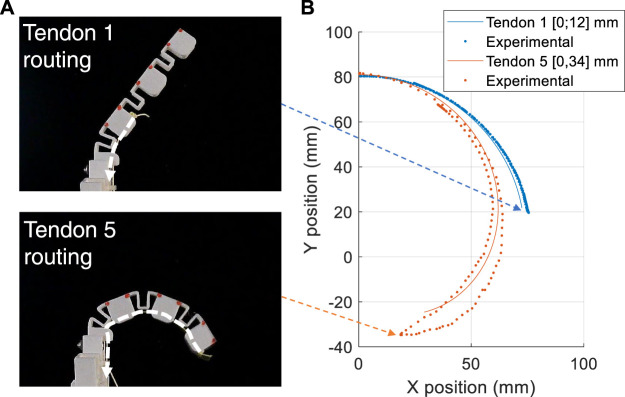
Finger model verification. **(A)** Pictures of one of the tested fingers. The red markers are applied for motion tracking purposes. In the top and bottom picture, respectively Tendon 1 and Tendon 5 are applied. **(B)** Comparison of the theoretical and experimental trajectories (maximum position error 8 mm).

To experimentally validate this approach, fingers with different tendon routing patterns were actuated and its position was recorded and compared to that of the model. For this example we consider only a single tendon. Figure 5A shows two fingers actuated by Tendon 1 and Tendon 5, respectively. In Figure 5B the theoretical-experimental trajectory comparison is presented. For the single tendon actuation (i.e.: referring to Figure 3A, only using Tendon 5), the routing lever (*ℓ*) has been fixed at to 8.5 mm. The model and experimental results show a close similarity, in particular so for the Tendon 1 motion. Both the experimental results show a hysteresis, which is more evident for Tendon 5 actuation where the range of deformation is greater. We hypothesis that this hysteresis occurs due to the friction in the system and some plastic deformation of the joints. Although this introduces some discrepancy into the results, the motion path and form of the model is highly similar to the experimental results—justifying the use of the proposed method for obtaining the inverse kinematics of a finger.

### 2.3 Combinatorial Actuation

To have complete control of each finger, 3 pairs of antagonistic tendons should be used. However, this is not scalable across the entire hand as it requires many additional actuators per finger, which must be controlled individually. To maintain a large workspace while keeping the actuator count low, we propose a new approach for the control and motion planning of the finger. In this approach we first actuate each finger with one front flexion tendon (Tendon 5) which can be controlled continuously. For the extension tendons, we use two discrete state or “digital” tendons for Tendon 2 and Tendon 4, which are shared across the entire hand. Hence, only two actuators control the extension tendons regardless of the number of fingers. When “on” these constrain the motion path of the finger and when “off” they are lose and do not affect the path of the finger. The digital tendons can be used combinatorially to produce four different trajectories in space by constraining the finger in different ways.

If a finger is actuated with only a single tendon (e.g., Tendon 5), its workspace is in fact a single line (with some hysteresis due to the friction between tendon and finger materials). By actuating the two digital tendons in the back side enables multiple single-line trajectories to be achieved. The single line trajectories form the “steady” workspace where the position of the fingertip can be maintained. The area between the steady workspace is the “transitory” workspace where the fingertip can reach the position when the digital tendons are switching.

**FIGURE 6:**
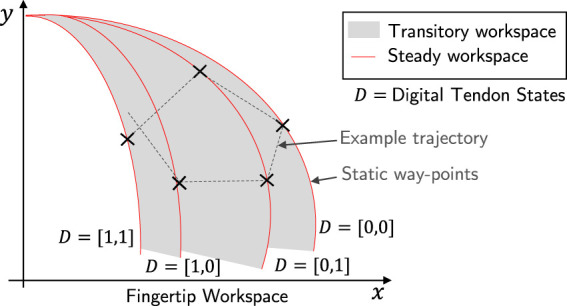
Combinatorial actuation concept. The finger is actuated by Tendon 5 (position-controlled) and Tendon 2 and 4 (digital). Four discrete trajectories (red lines) can then be obtained, whose points can be achieved for indefinite time. The other points (gray area) can only be reached during the transient due to the digital tendons switching.

Combinatorial actuation has the advantages of increasing the area of the workspace, and the ability to share this across fingers whilst still being able to use a simplified controller. The clear disadvantage is the lack of controllability within the workspace due to the transitory workspace where the pose cannot be held statically, and coupling in motion between fingers (further discussed in Section 2.5). Figure 6 shows the workspace of a combinatorially actuated single finger, with an example trajectory shown. Compared to a fully actuated finger, the combinatorially actuated finger is only guaranteed to reach points on the static way points, and the motion path between static waypoints are not guaranteed to be straight lines (the dotted lines on Figure 6 is only for illustration). Although this control approach may not be able to perform all complex tasks, we argue that for many cases this level of control is sufficient.

To leverage this approach we develop a controller and trajectory planner. The passive constraining behavior of the two back tendons can be built upon the existing finger model. Here “passive” means that the tendon does not perform any work on the structure. When “on”, it can theoretically transmit any force without moving (zero displacement condition); when “off”, it can freely move without transmitting force (zero force condition).

From our sampled kinematic correspondences (*Q*), we wish to extract samples of *Q* that can be achieved via combinatorial actuation. This corresponds to finding *Q* where the condition null work is applied on Tendon 2 and 4 (the two extension tendons), shown by 3, where *W*_*t*_ is a threshold value.

$$\text{Accept if: }|f_{2}\;q_{2}|<W_{t}\;\;\&\;\;|f_{4}\;q_{4}|<W_{t}$$ (3)

**FIGURE 7:**
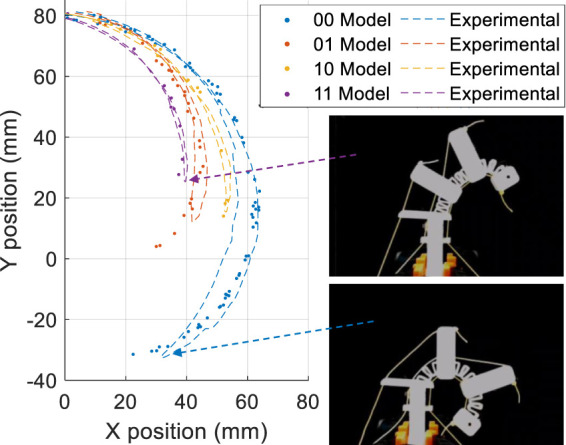
Trajectories extracted from the randomized workspace model compared to experimental trajectories (maximum position error 7 mm). In the legend the digital states are specified. The least significant digit refers to Tendon 2, the most significant digit to Tendon 4.

A validation of this model and experimental results are shown in Figure 7 when a combinatorial finger is actuated using the continuous flexure tendon with the different digital combinatorial states. Here we see the effect of the digital tendons in terms of constraining the motion to different areas of the workspace. There is a close correlation between the model and the experimental results.

### 2.4 Fingertip Workspace Optimization

A key advantage of our finger design is the ability to fabricate fingers with different geometries. This allows for tuning of the lever lengths of the digital tendons and the flexure joint compliances, to optimize the workspace. For practicality we fix the lever arm of the flexure tendon, but allow the levers for the extension tendons to extend outside the body. To design the optimal geometry we explored how the workspace of the finger tip varies for different finger geometries.

**FIGURE 8:**
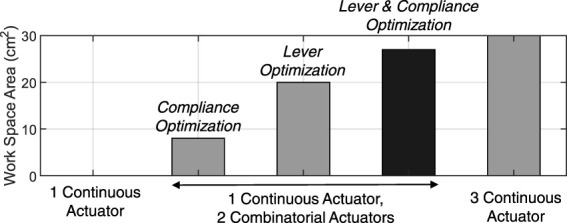
Comparison between the achievable workspace areas for different sets of optimal parameters.

We evaluated how the area of this workspaces varies when optimizing the joint compliances (keeping *ℓ* constant), the tendon levers *ℓ* (keeping the joints constant), and finally optimizing both. The optimization was performed by sampling uniformly over the search space and selecting the optimal configuration. For comparison, we also evaluated the maximum workspace area that can be obtained using continuous actuation for all three of the tendons. The results of our analysis (only performed by simulation) are represented in Figure 8 showing that by optimizing both compliance and levers the workspace becomes comparable (27 cm^2^) to a finger controlled with three active tendons while using a simple controller (30 cm^2^).

### 2.5 Trajectory Planning

Using the samples of reachable kinematic correspondences *Q*, we can then implement a motion planner for both single fingers and multiple fingers (see Figure 4 for the process of generating the trajectory). Starting from a single finger, given a generic point in space that we want to reach *P*_*des*_, the goal is to determine the digital states and tendon displacement to minimize the error *P*–*P*_*des*_ as such:

$$\left\{q_{f},D\right\}=arg\;min\|P-P_{\mathrm{des}}\|$$ (4)

where *q*_*f*_ is the displacement of the front tendon (Tendon 5), *D* = [*d*_0_, *d*_1_] is the vector of digital states, and ‖ ⋅‖ denotes the euclidean norm of a vector.

For motion planning of multiple fingers in a hand, the planner must consider the effect of the shared digital actuators across fingers. For some desired hand pose, the optimal digital state for a particular finger may be different to other fingers. Thus, we consider a multi-objective optimization problem, where we can weight the importance of each finger using a normalized vector of weights $w={\left[w_{1},\ldots ,w_{N}\right]}^{T}$ (*N* is the number of fingers). We now have a vector of desired points in space for each finger $P_{\mathrm{des}}={\left[P_{\mathrm{des,1}},\ldots ,P_{\mathrm{des,N}}\right]}^{T}$ and wish to find the tendon positions to minimize the overall position error as shown:

$$\left\{q_{f},D\right\}=arg\;min\overset{N}{\underset{i=1}{\sum }}w_{i}\|P_{i}-P_{\mathrm{des,i}}\|$$ (5)

where $q_{f}={\left[q_{f,1},\ldots ,q_{f,N}\right]}^{T}$ is the vector of front tendon displacements for each finger.

**FIGURE 9:**
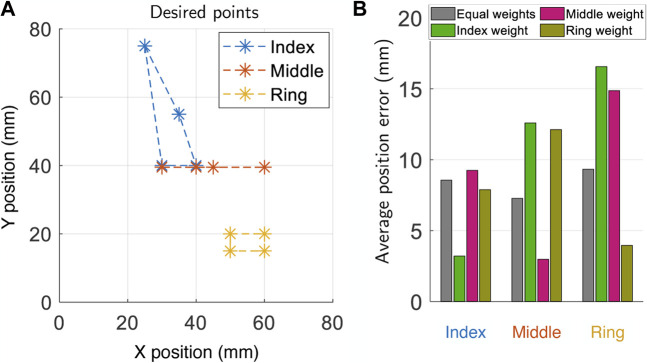
**(A)** Four-point trajectories used as test input for the multi-finger planner and **(B)** bar plot of the average error along the trajectory of each finger. The first weight set corresponds to equally weighted fingers, the following ones correspond to concentrating all the weight on a single finger among index, middle and ring finger.

The effect of the weights is visible for hand poses where each fingers have a different optimal digital state. To illustrate this point, consider the desired trajectory shown in Figure 9A. Here, the desired motion of each of the three fingers are sufficiently far apart in space and vary largely in its shape. This means, for each point of the trajectory, the optimal digital state for each finger is different. In Figure 9B we plot the average errors across the whole trajectories using four different settings of weights **w**. When the weight is tailored for a particular finger, the error is lowest for that finger, but high for others. Whereas, if equal weights are set for each finger, the error is approximately constant across all fingers.

### 2.6 Fingertip Sensing

Sensorized finger tips allow for improved closed loop manipulation and provide information about the environment. We wish to demonstrate the possibility of embedding sensors into the flexure hand in order to achieve more robust control in the future. In this work, we demonstrate how sensors could be incorporated and used by a simple closed loop controller. Taking inspiration from human mechanoreceptors, we designed a flexure based compliant fingertip through which runs a flexible tube (filled with air at standard room conditions) connected to a pressure transducer (MPXH6115AC6U). When a load is applied to the finger, the flexures compress and thus the flexible tube compresses, resulting in a measurable change in the internal air pressure. This fluidic sensing provides a mechanical method of sensing deformation which is inherently low drift and provide high sensitivity. This mechanism has been previously explored to provide tactile and strain sensing (Hughes et al., 2020; Tawk et al., 2020). The design of the compliant fingertips is shown in 10a; two 0.6 mm thick wave structures are used per side. The compliant structure is similar to the joint designs but has a symmetrical behavior to ensure the finger tips have stability. The fingertip can be printed in one-step as part of a whole finger, allowing a simple, quick and repeatable fabrication procedure.

**FIGURE 10:**
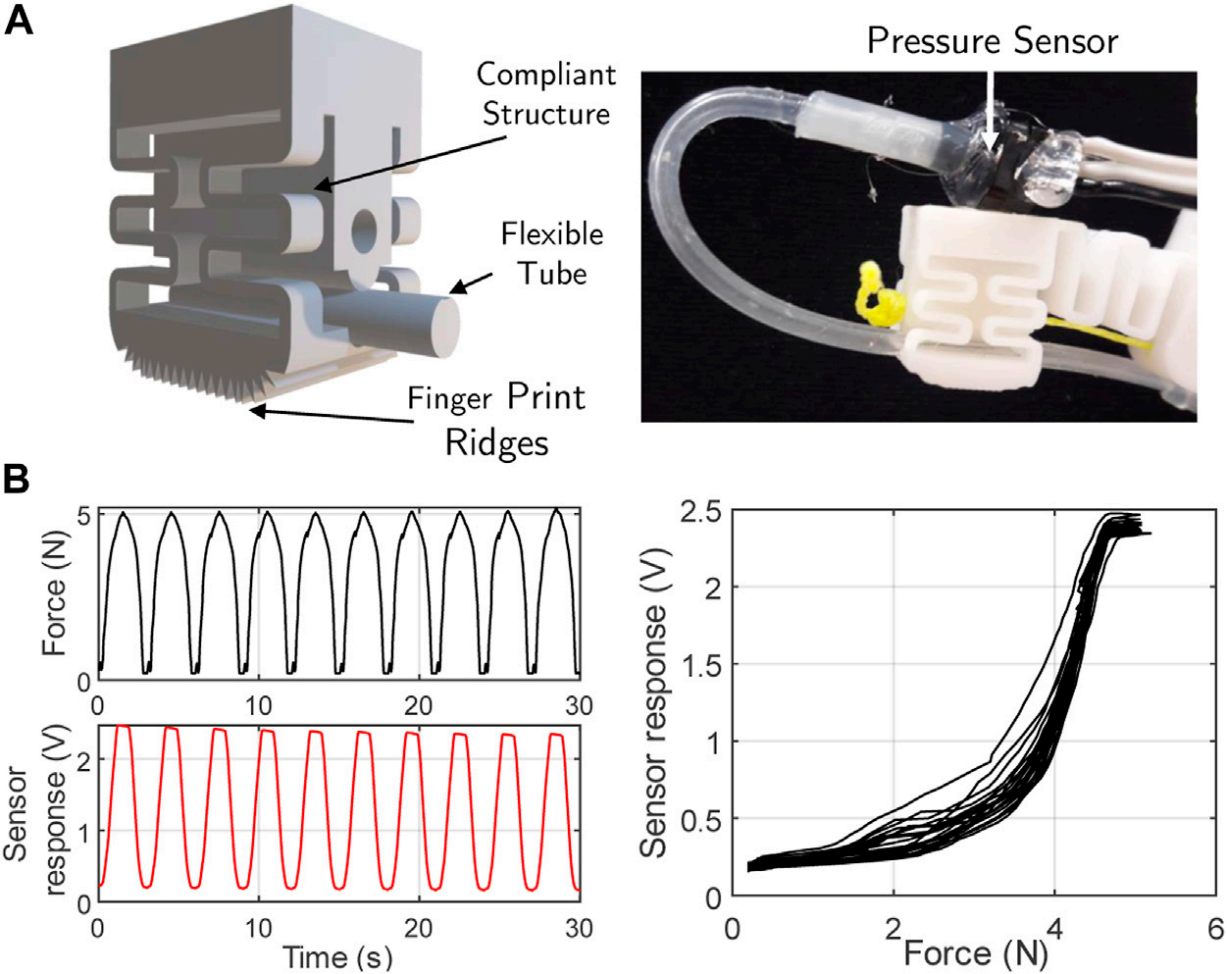
**(A)** Fingertip sensing system description and **(B)** plots of the applied force and the corresponding sensor response.

A typical force response of the fingertip under cyclic force loading is shown in Figure 10B as measured with a calibrated force testing rig. Although the response is not linear to force, it shows good sensitivity and repeatability. To aid with grasping the fingertips were printed with ridges to increase the friction and thus the grip on the grasped objects.

## 3 Experimental Setup

**FIGURE 11:**
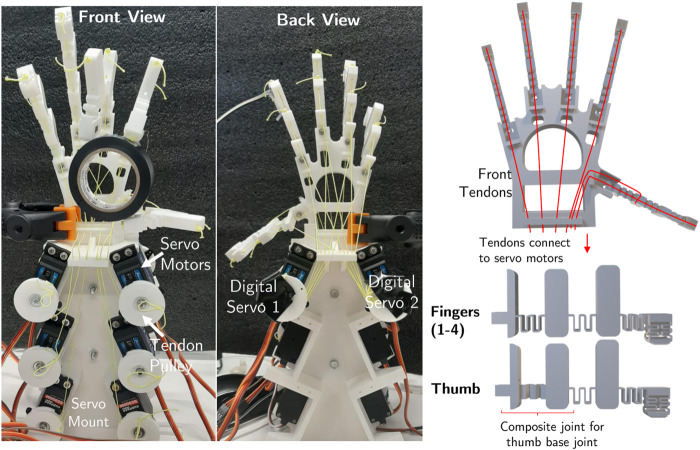
Photographs of the front and back view of the hand highlighting the digital tendon routing, and a diagram showing the tendon routing and designing of the fingers and thumbs of the hand.

The compliant fingers are printed in PLA using a Creality CR20 Pro printer. Each finger is printed individually and in an orientation such that the flexures are parallel to the print bed. This ensures the layers of the print run in the direction of bending, enabling the large deformations that occur in the joints. The four index fingers of the hand are scaled to reflex the sizes of human fingers and are attached to the main mount of the hand, where the angles and orientation of the finger mounted has been inspired by the human hand structure (Figure 11). To replicate the joint at the base of the thumb, which can be approximated as a ball and socket joint, a composite finger made from flexures in two orthogonal directions is used. The tendons for each finger are routed through the axial holes present in the links and then run through the hand mount and reach the servo motors. The thumb has an additional continuous tendon that provides an adduction motion, this routing is highlighted in Figure 11B. To actuate the tendons, servo motors are used with a 3D printed pulley attached to actuate each tendon. The hand is driven by six analog tendons (one for each finger and two for the thumb) and two digital tendons (acting on the four fingers simoultanously). The servo motors are controlled by a microcontroller using PWM, with the pressure sensor also connected to the ADC on the microcontroller. The full hand setup with the servos and tendons can be seen in Figure 11.

To quantitatively assess the finger trajectory control, a number of experiments were performed tracking the motion of the finger tips. This data was gathered recording videos of the sideways motion of the fingers, and using image detection tracking dots that were added to the sides of the fingers. The mapping from pixels to real-world coordinates can then be used to obtain the finger trajectory.

## 4 Results

### 4.1 Single Finger Trajectory Control

**FIGURE 12:**
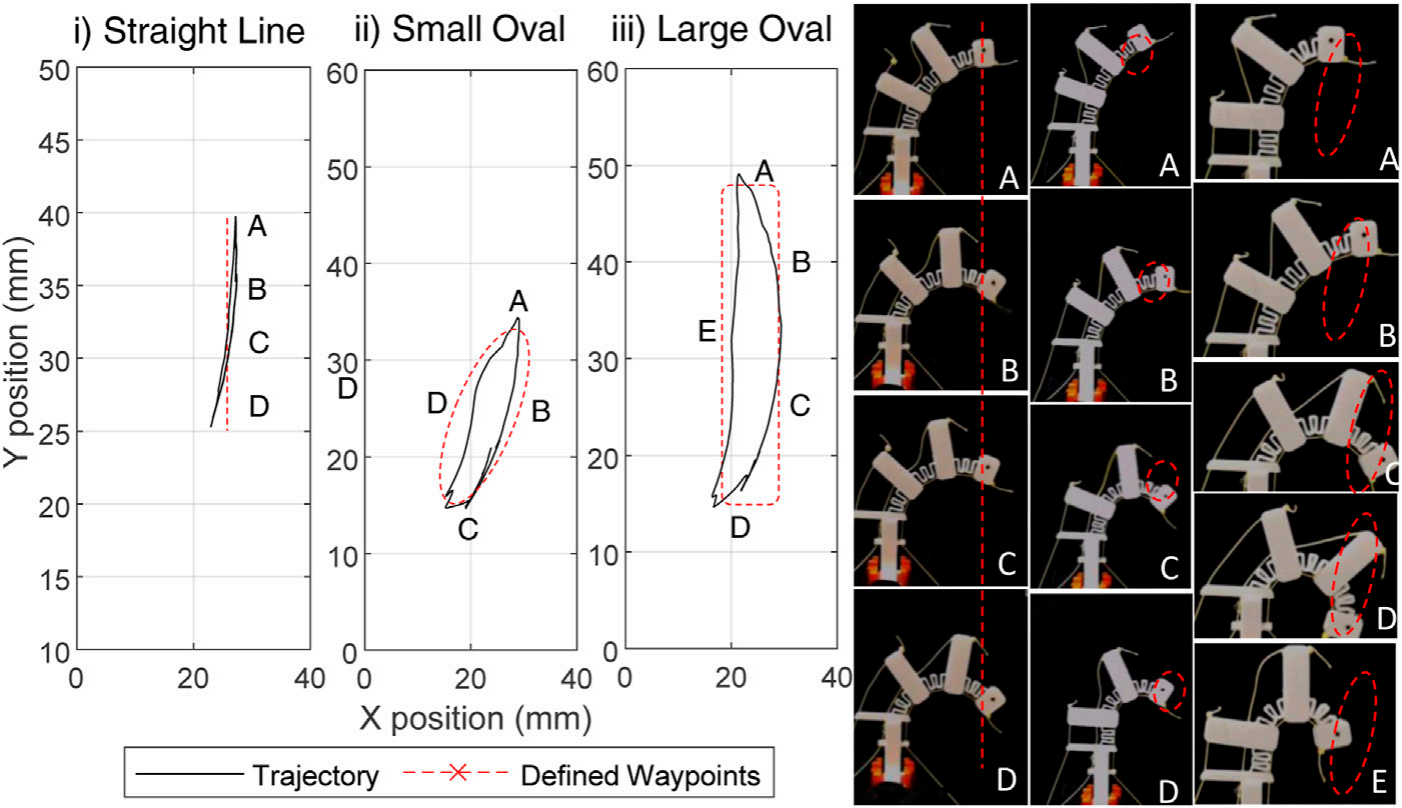
The motion of the finger tip in the workspace of a finger for three motions, showing both the real world trajectory and the defined motion. Images of the motion are shown to the right of the trajectories.

To demonstrate the single finger trajectory control a number example trajectories for performing a vertical motion and circular motion have been generated. These motions require the switching between digital states to achieve the motion. The tracked motion and finger poses are shown in Figure 12. While the motions are not “perfect,” they show a close similarity to the desired motion path. This highlights that whilst combinatorial actuation offers a compromise, it is still possible to have a degree of control over the motion of the finger tip.

### 4.2 Full Hand Physical Capabilities

**FIGURE 13:**
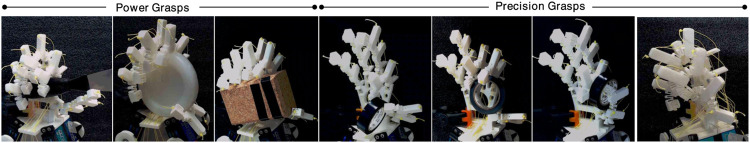
Example grasps based upon the grasp types from the Cutkosky hand grasp taxonomy (Cutkosky, 1989).

To demonstrate the physical capabilities of the hand and the range of motions and interactions offered by the thumb joints, a number of grasps have been demonstrated in Figure 13. These correspond to grasps from the Cutkosky grasp taxonomy (Cutkosky, 1989), showing both power and precision grasps. “Power grasps” are identified as those involving the full hand and where larger contact areas arise. On the other hand, in “precision grasps” the object is held by the thumb and the tips of other fingers. These grasps highlights the range of motion and dexterity of the thumb, with the hybrid orthogonal joint enables a number of complex grasp types. They also highlight how the workspace of the fingers enables a wide range of grasps, with the inherent compliance also offering some physical adaptation or robustness when grasping.

### 4.3 Multi-Finger Trajectory Control

**FIGURE 14:**
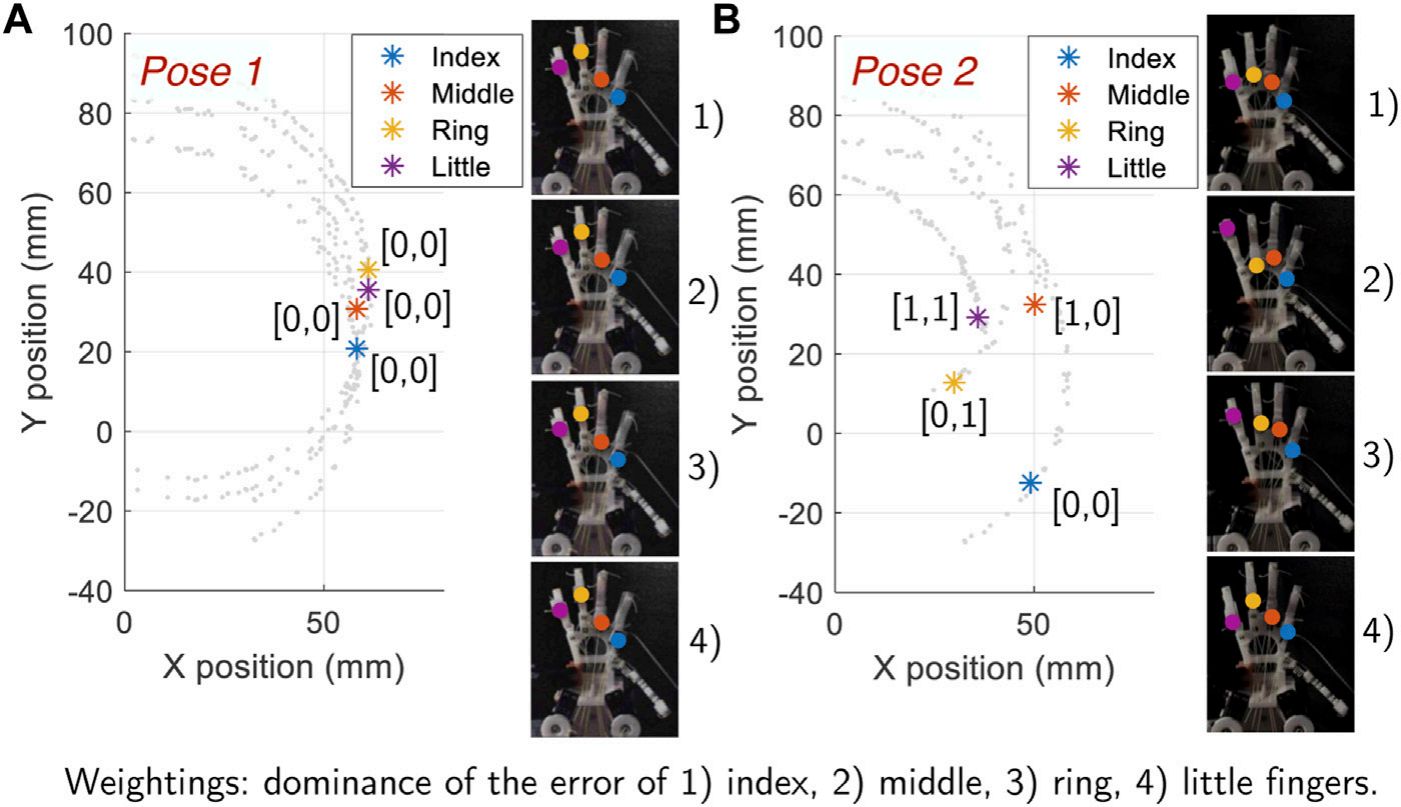
The effect of different weightings shown by using two different hand poses. **(A)** Pose 1: All fingers have the same optimal digital state, thus the pose does not vary by shifting the weights. **(B)** Pose 2: Each finger require a different digital state, hence the hand pose obtained differs by shifting the weights.

To explore trajectory control for multiple fingers two hand poses have been set. In pose 1, the optimal digital state for each finger is the same. In pose 2 the optimal digital state is different for all fingers. The plot in Figure 14A shows the desired finger position for pose 1. Since the optimal digital states are equivalent for each finger, even if the weights **w** are tailored for one particular finger, the same pose is achieved. This is not the case for pose 2, shown in Figure 14B. On the plot, we see that the desired hand position requires each finger to have a different optimal digital state. Therefore, as the weights are tuned to be tailored for a particular finger, although they are largely similar, the resulting hand pose changes (shown by the four hand pose images on the right hand side of the plot). Thus, for grasps where particular fingers are critical, those fingers should be given a higher weighting.

**FIGURE 15:**
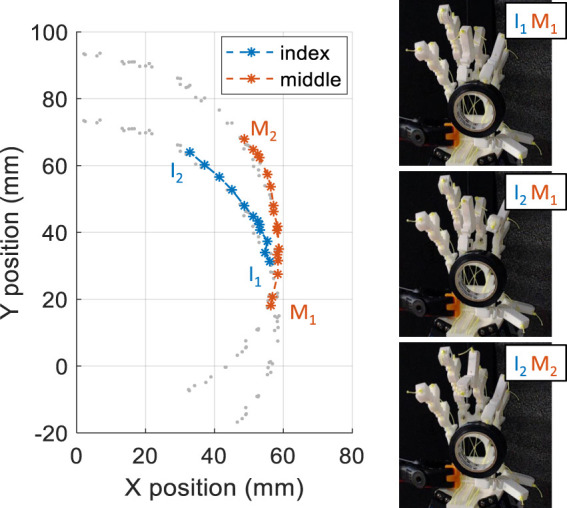
Demonstration of the multi-objective multi-finger control. A cylindrical object is held by two fingers while also exchanging fingers. The left plot shows the trajectory in the finger workspace, and the images on the right shows snapshots of the finger exchange in action. The hand is in $\left[0,0\right]$ state in this case.

To demonstrate the use of the multi-finger trajectory planning and control, the motion of holding a cylindrical object between the finger and thumb and then switching fingers while keeping the object stable, has been generated. This was performed by selecting the desired way points in the fingers work spaces and then using the trajectory planner to find the corresponding servo motor trajectories. The trajectories obtained are shown in Figure 15 alongside images highlighting the motion. This shows how combinatorial actuation and planner can allow multi-finger control for rapid and dexterous motion.

### 4.4 Hand Grasping Experiments

**FIGURE 16:**
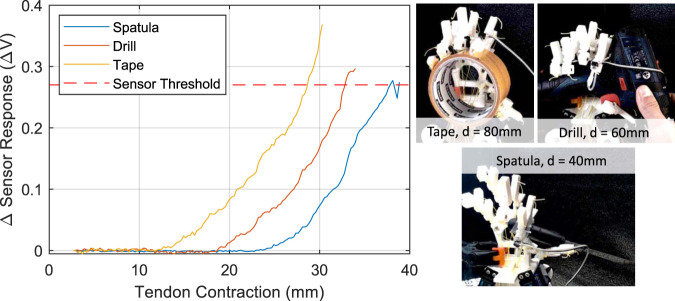
Left: Sensor response plotted against the tendon contraction of the index finger as objects are grasped. Right: The photographs of stable grasps with objects of varying size (diameter is noted).

The final experiment explores how the fingertip sensor can be combined with the trajectory planner to detect when a stable grasp is achieved. Here, a trajectory is chosen from the first two fingers (index and middle), and this trajectory is followed until a threshold in the sensor reading is exceeded. This allows to plan a trajectory (continuous tendons displacements and digital tendons combination) and stop the motion to limit the force exerted on the grasped object. Figure 16 shows stable grasp poses that are achievable and the corresponding sensor response from the fingertip where the threshold was found manually through experimentation. For the largest item the sensor response rises earlier, providing some indication of the size of the object.

## 5 Discussion

This paper presented a method of scalable fabrication and actuation for human inspired robotic hands. This is performed first by fabricating a hand using only FDM 3D printing by leveraging flexures for the joints and the compliant sensorized finger tips. By combining certain tendons on multiple fingers to be actuated at once, we are able to reduce the number of actuators required overall on the hand while having a sufficiently large workspace. While each finger has three tendons, we are able to limit the number of actuators to *n* + 2 rather than 3*n* where *n* is the number of fingers. To work with the combined tendons, we propose a combinatorial actuation method where we split the actuation on each finger to one continuous motion and a shared digital state. This actuation and control method does lead to a loss in precision in the motion tracking, and some dependency between fingers. However, when combined with the inherent compliance of the hand which offers some physical robustness the trajectory tracking is sufficiently accurate to perform complex grasps or motions, which are demonstrated through a range of full hand grasp motions.

A preliminary example of how the sensorized fingertips can be used to introduce closed-loop control into the combinatorial actuation model has also been presented. Further work is required to extend the combinatorial actuation approach to incorporate tactile feedback more centrally, to enable a wider variety of tasks. In addition, to improve the usability of this approach, the selection or identification of the way-points required for individual finger trajectories should be automated and streamlined.

## Data Availability

The raw data supporting the conclusion of this article will be made available by the authors, without undue reservation.
